# Dynamic frailty changes, cumulative frailty index, and the risk of stroke: Evidence from the China health and retirement longitudinal study

**DOI:** 10.1097/MD.0000000000049726

**Published:** 2026-07-10

**Authors:** Hui Li, Chuanyong Cui, Puchen Hong, Bi Tang

**Affiliations:** a Department of Cardiology, The First Affiliated Hospital of Bengbu Medical University, Bengbu, China; b Department of Clinical Medicine, Bengbu Medical University, Bengbu, China.

**Keywords:** CHARLS, cumulative frailty index, frailty state transition patterns, stroke

## Abstract

This study aimed to investigate the association between dynamic changes in frailty status, cumulative frailty index (FI), and the risk of incident stroke, providing novel evidence to inform stroke prevention strategies. This prospective study used data from the China health and retirement longitudinal study. Frailty transitions across 2 waves were classified into 7 patterns: stable robust, robust to pre-frail/frail, stable pre-frail, pre-frail to robust, pre-frail to frail, stable frail, and frail to pre-frail/robust. Multivariable Cox models estimated stroke risk for each transition, with “stable robust” as the reference. The cumulative FI was assessed categorically (quartiles) and continuously (per standard deviation increase). Restricted cubic splines evaluated dose–response associations. Subgroup and sensitivity analyses tested robustness. Among 6947 participants (median follow-up 7 years), stroke risk varied markedly by frailty transition. Compared with stable robust, the stable frail group showed the highest risk (hazard ratio [HR] = 4.48; 95% confidence interval [CI]: 3.25–6.18). Robust to pre-frail/frail transitions increased risk by 81% (HR = 1.81; 95% CI: 1.31–2.50). Relative to stable pre-frail, improving to robust was protective, whereas transition to frail increased risk by 58% (HR = 1.58; 95% CI: 1.24–2.01). Compared with stable frail, improvement to pre-frail/robust reduced risk (HR = 0.60; 95% CI: 0.43–0.84). The cumulative FI showed a strong linear association with stroke: each 1 − standard deviation increase was associated with a 55% higher risk (HR = 1.55; 95% CI: 1.45–1.65), and participants in the highest versus lowest quartile had over 3-fold higher risk (HR = 3.29; 95% CI: 2.59–4.17). Worsening frailty status substantially elevates stroke risk, whereas frailty improvement confers measurable protection. Cumulative FI is linearly associated with incident stroke, underscoring the importance of early identification and long-term management of frailty in stroke prevention.

## 1. Introduction

As global populations age, frailty has become a major focus in geriatric and cardiovascular research. Defined as a clinical phenotype of biological aging, frailty is characterized by diminished physiological reserves, multisystem impairments, and heightened vulnerability to stressors.^[1,2]^ A substantial body of evidence identifies frailty as a powerful predictor of adverse health outcomes – including mortality, disability, and hospitalization^[3]^ – and links it to a broad spectrum of chronic diseases.^[4]^ Epidemiological data further indicate that approximately 10% of community-dwelling older adults are frail,^[5]^ underscoring its growing public health relevance.

Stroke remains one of the leading causes of death and disability worldwide. The 2019 Global Burden of Disease Study reported nearly 12.2 million incident cases and 6.6 million stroke-related deaths globally,^[6]^ highlighting persistent challenges in prevention and long-term management.^[7]^ Older adults, who face the highest stroke risk, also exhibit the greatest frailty burden, resulting in considerable overlap between the 2 conditions. Physiopathologic mechanism studies suggest that frailty may increase stroke susceptibility through dysregulated inflammatory responses,^[8]^ endothelial dysfunction,^[9]^ and impaired physiological resilience. Multiple epidemiological studies provide evidence of a strong association between frailty and stroke.^[10,11]^

However, the contribution of frailty indicators to primary stroke prevention remains incompletely defined. Previous studies have primarily relied on cross-sectional assessments or trajectory-based classifications, approaches that may not fully capture clinically meaningful changes in frailty status over time. For example, the cross-sectional study by Zhang et al was unable to evaluate the temporal association between frailty and subsequent stroke risk,^[10]^ whereas the trajectory-based analysis by Song et al did not directly examine how transitions between specific frailty states were associated with stroke risk according to baseline frailty status.^[12]^ This distinction is clinically important, as frailty is a dynamic and potentially reversible condition; its worsening or improvement may reflect changes in physiological reserve, systemic inflammation, vascular function, and resilience.^[13]^ Moreover, stroke risk may be shaped not only by frailty measured at a single time point but also by the cumulative burden of frailty exposure, which may more accurately capture sustained physiological vulnerability and progressive deficit accumulation. Nevertheless, few population-based studies have simultaneously investigated frailty transitions and cumulative frailty burden in relation to incident stroke.

To address these limitations, the present study systematically examines the association between dynamic frailty transitions and incident stroke risk and introduces a cumulative FI to quantify long-term frailty burden. These findings aim to provide novel evidence to support early risk stratification and precision prevention strategies for stroke.

## 2. Methods

### 2.1. Data source and study population

This study analyzed publicly available data from the China health and retirement longitudinal study (CHARLS), a nationally representative cohort initiated in 2011. CHARLS employs a multistage, stratified, probability proportional to size sampling design, covering 450 villages or residential communities across 150 counties in 28 provinces. Biennial follow-up surveys collect detailed information on physical and mental health, sociodemographic characteristics, and family structure among adults aged ≥45 years, with response rates consistently exceeding 80%. The study protocol was approved by the Biomedical Ethics Review Committee of Peking University (IRB00001052-11015), and all participants provided written informed consent.^[14]^ Reporting complies with the Strengthening the Reporting of Observational Studies in Epidemiology guidelines.^[15]^

Frailty state transition patterns and cumulative FI were assessed using Wave 1 (2011) and Wave 2 (2013) data, which together defined the baseline period. Incident stroke events were identified during follow-up in Waves 3 (2015), 4 (2018), and 5 (2020).

Participants were eligible if they completed both Waves 1 and 2 with complete baseline information. Exclusion criteria included: individuals who have <30 variable responses required for the calculation of weakness indicators in Wave 1 or 2; self-reported physician-diagnosed stroke in Wave 1 or 2; and age <45 years. The participant selection process is shown in Figure S1, Supplemental Digital Content 1.

### 2.2. Assessment of frailty state transition patterns and cumulative frailty index (FI)

Frailty was quantified using a 35-item FI based on the Rockwood–Mitnitski deficit accumulation model,^[16]^ incorporating limitations in activities of daily living and instrumental activities of daily living, self-reported chronic diseases, psychological distress, and self-rated health (Table S1, Supplemental Digital Content 6).^[17]^ FI values were categorized according to established thresholds: robust (FI ≤ 0.10), pre-frail (0.10 < FI < 0.25), and frail (FI ≥ 0.25).^[18]^ Transitions between Waves 1 and 2 were classified into 7 distinct patterns: stable robust; robust to pre-frail/frail; stable pre-frail; pre-frail to robust; pre-frail to frail; stable frail; and frail to pre-frail/robust.

To capture the long-term burden of frailty, a cumulative FI was derived using the following formula:


$$Cumulative\;\;\;FI=\left(\frac{FI_{Wave1}+FI_{Wave2}}{2}\right)\times time\;\;\;interval\;\;\;(years)$$

This measure reflects both the average frailty level and the duration of exposure across the 2-year baseline window, thereby providing a more integrated representation of sustained physiological decline.^[19]^

### 2.3. Assessment of outcome and follow-up time

Incident stroke was identified based on participants self-report of a physician diagnosis (“Has a doctor ever told you that you have had a stroke?”).^[20]^ Follow-up commenced at the end of the baseline period in 2013, with duration calculated as follows. First, for participants without stroke, follow-up duration was calculated as the time of their last survey participation minus the end of the baseline period (2013). Second, for participants lost to follow-up, follow-up duration was approximated as the integer difference between the year of their last survey and 2013. Third, for participants who reported a new stroke during follow-up: if the 1st stroke diagnosis was documented in the *k*th wave (*k* = 3, 4, or 5), the event was assumed to occur at the midpoint between the *k*th and (*k* − 1)th waves. The calculation formula is: follow-up duration = [(*k*th wave survey year − (*k* − 1)th wave survey year)/2] + [(*k* − 1)th wave survey year − 2013]. Fourth, for individuals with unknown stroke onset time, the estimated event time was the midpoint between the survey time of the 1st stroke report and that of the preceding wave. Approximate follow-up duration was then calculated relative to the end of the baseline period. This midpoint-assignment strategy reduces potential misclassification arising from the interval-censored nature of CHARLS and provides a more accurate estimation of follow-up time within the constraints of biennial follow-up.^[19]^

### 2.4. Covariate assessment

Potential confounders were identified using a directed acyclic graph (www.dagitty.net), which informed the selection of the minimal sufficient adjustment set to minimize confounding bias and clarify the key variables along the causal pathway^[21]^ (Fig. S2, Supplemental Digital Content 2). Based on the directed acyclic graph analysis, the covariates requiring adjustment included age, sex, body mass index (BMI), smoking status, drinking status, hypertension, dyslipidemia, diabetes mellitus (DM), and heart disease.

Sociodemographic characteristics included age and sex. BMI was calculated as weight divided by height squared (kg/m^2^). Lifestyle variables consisted of smoking and drinking use: smoking status was categorized as never or ever smoker (including former and current smokers), and drinking status use as never or ever drinker (including former and current drinker) based on self-report. Chronic disease variables were defined as follows: DM was defined by self-reported physician diagnosis, fasting glucose ≥7.0 mmol/L, non-fasting glucose ≥11.1 mmol/L, or glycated hemoglobin ≥6.5%; hypertension was defined as self-reported diagnosis or a mean systolic blood pressure ≥140 mm Hg or diastolic blood pressure ≥90 mm Hg; dyslipidemia was defined as a self-reported hyperlipidemia or high-density lipoprotein cholesterol <1.0 mmol/L; heart disease was based on self-reported physician-diagnosed cardiovascular conditions.

For missing data, continuous variables were imputed using median values, while categorical variables were assigned a separate “missing” category to retain sample size and reduce potential bias arising from incomplete data.

### 2.5. Statistical analysis

Baseline characteristics were summarized according to frailty-transition patterns. Continuous variables were presented as means ± standard deviations (SD), and categorical variables as counts and percentages.

To evaluate the associations of frailty state transition patterns and the cumulative FI with incident stroke, hazard ratios (HRs) and 95% confidence intervals (95% CIs) were estimated using Cox proportional hazards models. Proportional hazards assumptions were assessed using Schoenfeld residuals, and no violations were detected (*P* > .05), indicating adequate model fit. Three sequential models were then constructed: a crude model without covariate adjustment; model 1, adjusted for age, sex, BMI, smoking status, and drinking status; and model 2, further adjusted for hypertension, dyslipidemia, diabetes, and a history of heart disease.

The primary analyses consisted of 2 components. First, using the “stable robust” group as the reference, Cox models were fitted to estimate stroke risk across all 7 frailty-transition patterns. Second, these patterns were further classified into 3 groups based on baseline frailty status: group 1 (stable robust and robust to pre-frail/frail), group 2 (stable pre-frail; pre-frail to robust; and pre-frail to frail), and group 3 (stable frail and frail to pre-frail/robust). For each group, the stable frailty state (stable robust, stable pre-frail, or stable frail) served as the reference to evaluate how different transition directions influenced stroke risk.

For analyses involving the cumulative FI, participants were categorized into quartiles, with the lowest quartile serving as the reference, and the standard deviation regression model was also included for analysis simultaneously. Linear trends were tested using the median value of each quartile. Restricted cubic spline models were further applied to explore potential nonlinear dose–response relationships between the cumulative FI and stroke risk.

Subgroup analyses were performed stratified by age (<60 vs ≥60 years), sex, smoking status, drinking status, DM, hypertension, dyslipidemia, and heart disease. Effect modification was assessed using interaction terms in the Cox models.

To assess the robustness of the findings, 3 sensitivity analyses were conducted: expanding the covariate set to include marital status, education, physical activity, high-density lipoprotein cholesterol, C-reactive protein, glycated hemoglobin, mean systolic blood pressure, and mean diastolic blood pressure; excluding individuals who developed stroke at the 3rd-wave follow-up to minimize potential reverse causation; and performing multiple imputation for missing covariates and combining estimates using Rubin rules.

Exploratory age-stratified analyses were further conducted to provide dedicated risk stratification for older participants (<75 vs ≥75 years) and to assess whether the associations of frailty state transition patterns and cumulative FI with incident stroke differed in the older age subgroup.

All statistical analyses were conducted using R software (version 4.5.1; R Foundation for Statistical Computing, Vienna, Austria), and 2-sided *P* values <.05 were considered statistically significant.

## 3. Results

A total of 6947 participants were included in the analysis. Baseline characteristics are summarized in Table 1. The mean age was 61.19 years (SD 9.72), and 61.95% were women. Participants in the “stable frail” group were notably older (65.81 ± 10.65 vs 58.76 ± 9.34 years) and had a higher proportion of women (66.73% vs 56.96%) compared with those who were stable robust. The overall mean BMI was 23.56 kg/m^2^ (SD 3.82). At baseline, the prevalence of DM, hypertension, dyslipidemia, and heart disease was 14.02%, 43.60%, 34.01%, and 16.48%, respectively. The “stable frail” group had markedly higher prevalences of DM (17.44% vs 12.45%), hypertension (50.89% vs 41.63%), heart disease (38.97% vs 2.72%), and a lower prevalence of drinking status (30.78% vs 39.22%) compared with the “stable robust” group. The remaining frailty-transition groups exhibited baseline profiles generally falling between these 2 extremes.

**Table 1:** Baseline characteristics of participants according to frail state transition pattern.

	Total	Stable robust	Pre-frail to robust	Robust to pre-frail/frail	Stable pre-frail	Frail to pre-frail/robust	Pre-frail to frail	Stable frail
Sample size	6947	1285	719	972	2393	411	605	562
Age	61.19 ± 9.72	58.76 ± 9.34	59.86 ± 9.32	60.56 ± 9.61	60.92 ± 9.22	64.15 ± 9.63	63.68 ± 9.83	65.81 ± 10.65
Sex								
Missing	5 (0.07)	2 (0.16)	1 (0.14)	1 (0.10)	1 (0.04)			
Female	4304 (61.95)	732 (56.96)	444 (61.75)	598 (61.52)	1494 (62.43)	265 (64.48)	396 (65.45)	375 (66.73)
Male	2638 (37.97)	551 (42.88)	274 (38.11)	373 (38.37)	898 (37.53)	146 (35.52)	209 (34.55)	187 (33.27)
Smoking status								
Never smokers	4580 (65.93)	827 (64.36)	481 (66.90)	637 (65.53)	1578 (65.94)	283 (68.86)	407 (67.27)	367 (65.30)
Ever smokers	2367 (34.07)	458 (35.64)	238 (33.10)	335 (34.47)	815 (34.06)	128 (31.14)	198 (32.73)	195 (34.70)
Drinking status								
Never drinkers	4490 (64.63)	781 (60.78)	455 (63.28)	629 (64.71)	1558 (65.11)	274 (66.67)	404 (66.78)	389 (69.22)
Ever drinkers	2457 (35.37)	504 (39.22)	264 (36.72)	343 (35.29)	835 (34.89)	137 (33.33)	201 (33.22)	173 (30.78)
BMI	23.56 ± 3.82	24.02 ± 3.60	23.73 ± 4.16	23.58 ± 3.75	23.48 ± 3.98	23.17 ± 3.65	23.20 ± 3.82	23.28 ± 3.31
DM								
Missing	10 (0.14)	1 (0.08)	1 (0.14)		5 (0.21)		1 (0.17)	2 (0.36)
No	5963 (85.84)	1124 (87.47)	629 (87.48)	826 (84.98)	2052 (85.75)	342 (83.21)	528 (87.27)	462 (82.21)
Yes	974 (14.02)	160 (12.45)	89 (12.38)	146 (15.02)	336 (14.04)	69 (16.79)	76 (12.56)	98 (17.44)
Hypertension								
Missing	3 (0.04)	2 (0.08)					1 (0.18)	
No	3915 (56.36)	750 (58.37)	439 (61.06)	556 (57.20)	1362 (56.92)	202 (49.15)	331 (54.71)	275 (48.93)
Yes	3029 (43.60)	535 (41.63)	280 (38.94)	416 (42.80)	1029 (43.00)	209 (50.85)	274 (45.29)	286 (50.89)
Dyslipidemia								
Missing	40 (0.58)	1 (0.08)	8 (1.11)	7 (0.72)	13 (0.54)	4 (0.97)	3 (0.50)	4 (0.71)
No	4544 (65.41)	845 (65.76)	495 (68.85)	625 (64.30)	1556 (65.02)	275 (66.91)	398 (65.79)	350 (62.28)
Yes	2363 (34.01)	439 (34.16)	216 (30.04)	340 (34.98)	824 (34.43)	132 (32.12)	204 (33.72)	208 (37.01)
Heart disease								
Missing	34 (0.49)	2 (0.16)	6 (0.83)	3 (0.31)	13 (0.54)	2 (0.49)	4 (0.66)	4 (0.71)
No	5768 (83.03)	1248 (97.12)	661 (91.93)	876 (90.12)	1888 (78.90)	294 (71.53)	462 (76.36)	339 (60.32)
Yes	1145 (16.48)	35 (2.72)	52 (7.23)	93 (9.57)	492 (20.56)	115 (27.98)	139 (22.98)	219 (38.97)

BMI = body mass index, DM = diabetes mellitus.

### 3.1. Frailty-transition patterns and incident stroke in the whole cohort

The associations between the 7 frailty-transition patterns and incident stroke, using the “stable robust” group as the reference, demonstrated a clear and progressive increase in risk across transition categories, as shown in Table 2. Participants transitioning from robust to pre-frail/frail had an HR of 1.73 (95% CI: 1.25–2.38); those stable pre-frail had an HR of 2.16 (95% CI: 1.65–2.83); those transitioning from frail to pre-frail/robust had an HR of 2.70 (95% CI: 1.87–3.90); those transitioning from pre-frail to frail had an HR of 3.44 (95% CI: 2.49–4.74); and those stable frail had the highest risk (HR: 4.48; 95% CI: 3.25–6.18). By contrast, the “pre-frail to robust” group did not show a statistically significant reduction in risk. The HRs in the crude model and model 1 were identical (1.31; 95% CI: 0.91–1.90; *P* = .15), and the HR increased slightly in model 2 (1.38; 95% CI: 0.95–2.00), approaching but not achieving statistical significance.

**Table 2:** Associations of the frail state transition pattern with stroke, evaluated using the Cox proportional hazards model.

Exposure	Crude model		Model 1		Model 2	
	HR (95% CI)	*P* value	HR (95% CI)	*P* value	HR (95% CI)	*P* value
Stable robust	Ref.		Ref.		Ref.	
Pre-frail to robust	1.31 (0.91–1.90)	.15	1.31 (0.91–1.90)	.15	1.38 (0.95–2.00)	.09
Robust to pre-frail/frail	1.72 (1.25–2.37)	<.001	1.74 (1.26–2.40)	<.001	1.73 (1.25–2.38)	<.001
Stable pre-frail	2.16 (1.66–2.83)	<.001	2.18 (1.67–2.85)	<.001	2.16 (1.65–2.83)	<.001
Frail to pre-frail/robust	2.78 (1.94–3.98)	<.001	2.78 (1.94–3.99)	<.001	2.70 (1.87–3.90)	<.001
Pre-frail to frail	3.40 (2.48–4.67)	<.001	3.46 (2.52–4.76)	<.001	3.44 (2.49–4.74)	<.001
Stable frail	4.77 (3.51–6.48)	<.001	4.66 (3.41–6.37)	<.001	4.48 (3.25–6.18)	<.001
P for trend		<.001		<.001		<.001

Crudel model: No covariates were adjusted.

Model 1: Age, sex, smoke status, drink status, and BMI.

Model 2: Age, sex, smoke status, drink status, BMI, DM, hypertension, dyslipidemia, and heart disease.

BMI = body mass index, CI = confidence interval, DM = diabetes mellitus, HR = hazard ratio.

### 3.2. Stratified analysis by levels of frailty

When analyses were stratified by different levels of frailty status, the associations remained consistent. Transitions to worse frailty states were linked to progressively higher stroke risk, whereas improvement in frailty was associated with lower risk, as shown in Table 3. Among participants whose initial frailty state was robust, the transition from robust to pre-frail/frail was associated with progressively higher risk across models (crude HR: 1.72; model 1 HR: 1.75; model 2 HR: 1.81). Among participants whose initial frailty state was pre-frail, reversion to robustness was consistently associated with lower stroke risk, with HRs of 0.61, 0.60, and 0.65 across the 3 models. In contrast, transitioning from pre-frail to frail resulted in a stable elevation in stroke risk, with HRs consistently around 1.58. Among participants whose initial frailty state was frail, improvement in frailty status (transitioning to pre-frail or robust) was associated with a substantially lower risk of stroke, with HRs of 0.59, 0.60, and 0.60 across the 3 models.

**Table 3:** Associations of the frail state transition pattern with stroke, evaluated using the Cox proportional hazards model.

Exposure	HR (95% CI)	*P* value	HR (95% CI)	*P* value	HR (95% CI)	*P* value
The 1st group						
Stable robust	Ref.		Ref.		Ref.	
Robust to pre-frail/frail	1.72 (1.25–2.37)	<.001	1.75 (1.27–2.42)	<.001	1.81 (1.31–2.50)	<.001
The 2nd group						
Stable pre-frail	Ref.		Ref.		Ref.	
Pre-frail to robust	0.61 (0.45–0.82)	.001	0.6 (0.44–0.82)	.001	0.65 (0.48–0.89)	.01
Pre-frail to frail	1.58 (1.24–2.01)	<.001	1.57 (1.23–2.00)	<.001	1.58 (1.24–2.01)	<.001
The 3rd group						
Stable frail	Ref.		Ref.		Ref.	
Frail to pre-frail/robust	0.59 (0.42–0.82)	.002	0.60 (0.43–0.83)	.002	0.60 (0.43–0.84)	.003

Crudel model: No covariates were adjusted.

Model 1: Age, sex, smoke status, drink status, and BMI.

Model 2: Age, sex, smoke status, drink status, BMI, DM, hypertension, dyslipidemia, and heart disease.

BMI = body mass index, CI = confidence interval, DM = diabetes mellitus, HR = hazard ratio.

### 3.3. Cumulative FI and stroke risk

**Figure 1.**
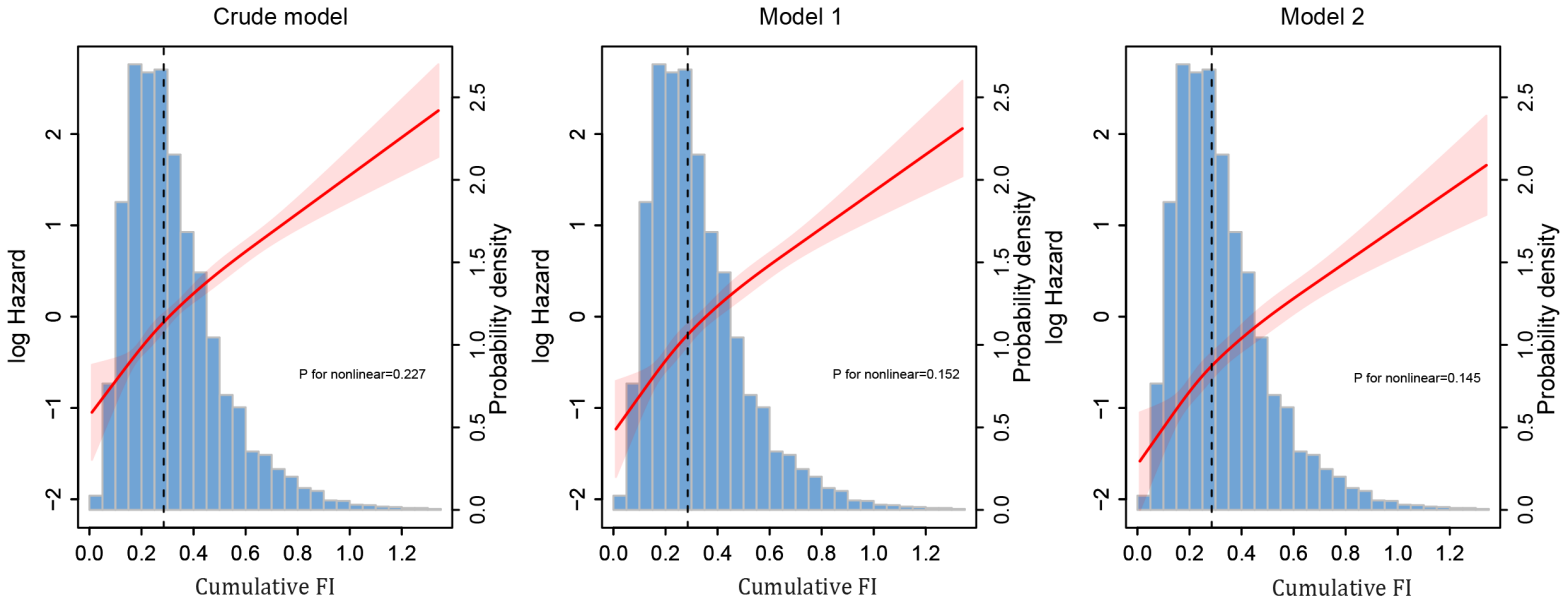
Dose–response relationship between the cumulative frailty index and incident stroke. Restricted cubic spline (RCS) analyses were performed to explore the association between the cumulative frailty index and incident stroke across 3 adjusted models. A stable linear positive association was observed in all models (*P* for nonlinearity >.05 for all). Model adjustments included: crude model (no covariate adjustment); model 1 (adjusted for age, sex, BMI, smoking status, and drinking status); and model 2 (further adjusted for DM, hypertension, dyslipidemia, and heart disease). BMI = body mass index, DM = diabetes mellitus, FI = frailty index.

When the cumulative FI was categorized into quartiles, a pronounced stepwise increase in stroke risk was observed. Compared with the lowest quartile (*Q*1), the crude HRs were 1.37 for *Q*2, 1.96 for *Q*3, and 3.43 for *Q*4. These associations remained largely unchanged after adjustment for demographic and lifestyle factors (model 1). In model 2, which further adjusted for chronic disease burden, the HRs showed slight attenuation but preserved the overall pattern: 1.36 for *Q*2, 1.95 for *Q*3, and 3.29 for *Q4*. Trend tests based on quartile medians were statistically significant in all models (*P* < .001). Meanwhile, for every 1 − SD increase in the cumulative FI, the risk increased by 55% (Table 4). Restricted cubic spline analyses (Fig. 1) supported a linear relationship between cumulative FI and stroke risk. Nonlinearity tests yielded *P* values of .227, .152, and .145 in the 3 models, indicating no evidence of deviation from linearity and confirming a stable positive linear association.

**Table 4:** Associations of the cumulative frailty index with stroke, evaluated using the Cox proportional hazards model in the whole cohort.

Exposure	Crude model		Model 1		Model 2	
	HR (95% CI)	*P* value	HR (95% CI)	*P* value	HR (95% CI)	*P* value
Per 1 − SD increase	1.57 (1.48–1.67)	<.001	1.57 (1.47–1.67)	<.001	1.55 (1.45–1.65)	<.001
Quatipartiple group						
Q1	Ref.		Ref.		Ref.	
Q2	1.37 (1.06–1.77)	.02	1.37 (1.06–1.78)	.02	1.36 (1.05–1.77)	.02
Q3	1.96 (1.54–2.49)	<.001	1.98 (1.55–2.52)	<.001	1.95 (1.53–2.49)	<.001
Q4	3.43 (2.73–4.31)	<.001	3.43 (2.72–4.32)	<.001	3.29 (2.59–4.17)	<.001
*P* for trend		<.001		<.001		<.001
*P* for trend (median value)		<.001		<.001		<.001

Crudel model: No covariates were adjusted.

Model 1: Age, sex, smoke status, drink status, and BMI.

Model 2: Age, sex, smoke status, drink status, BMI, DM, hypertension, dyslipidemia, and heart disease.

BMI = body mass index, CI = confidence interval, DM = diabetes mellitus, HR = hazard ratio.

### 3.4. Subgroup and sensitivity analyses

Subgroup analyses (Figs. S3–S5, Supplemental Digital Content 3) demonstrated that the positive association between frailty transitions and stroke risk was largely consistent across categories of age, sex, lifestyle factors, and cardiometabolic comorbidities. A significant interaction was observed only among participants with heart disease. The findings remained robust across multiple sensitivity analyses. Adjustment for an expanded set of covariates (Tables S2–S4, Supplemental Digital Content 7), exclusion of participants who experienced stroke at the 3rd-wave follow-up (Tables S5–S7, Supplemental Digital Content 10), and the application of multiple imputation for missing covariates (Tables S8–S10, Supplemental Digital Content 13) all yielded results similar to the primary analyses.

### 3.5. Exploratory age-stratified analyses

In exploratory analyses stratified by age (<75 vs ≥75 years), the associations of frailty-transition patterns with stroke risk among participants aged <75 years were largely consistent with the primary findings. Among participants aged ≥75 years, the associations were generally directionally concordant; however, statistical evidence was weaker, with fewer associations reaching significance and wider CIs, consistent with the smaller number of participants and stroke events in this subgroup. In model 2, only progression from pre-frail to frail status reached statistical significance, both relative to stable robust status (HR: 3.43; 95% CI: 1.14–10.32) (Tables S11, Supplemental Digital Content 16) and stable pre-frail status (HR: 2.66; 95% CI: 1.16–6.08) (Tables S12, Supplemental Digital Content 17).

The cumulative FI was consistently associated with stroke risk across age strata (Tables S13, Supplemental Digital Content 18). Each 1 − SD increment in cumulative FI was associated with a higher risk of stroke among participants aged <75 years (HR: 1.56; 95% CI: 1.46–1.67) and ≥75 years (HR: 1.55; 95% CI: 1.25–1.94). Stroke risk increased progressively across quartiles of cumulative FI in both age groups, with significant linear trends among participants aged <75 and ≥75 years (both *P* for trend <.001). Quartile-specific associations in the ≥75-year subgroup showed the same overall pattern, although fewer estimates reached statistical significance and CIs were wider.

## 4. Discussion

In this prospective cohort study, we observed significant and independent associations between dynamic frailty transitions and the risk of stroke. As individuals progressed from “stable robust” to “stable frail,” stroke risk rose in a clear stepwise pattern. Conversely, improvement in frailty – regardless of initial status – was consistently associated with a reduction in stroke risk, underscoring frailty as a biologically modifiable condition whose reversal may yield substantial cerebrovascular benefits. In parallel, the cumulative FI showed a strong linear relationship with incident stroke, with risk increasing steadily as long-term frailty burden accumulated. Together, these findings indicate that both short-term deterioration and chronic physiologic wear reflected by cumulative frailty contribute meaningfully to stroke susceptibility.

Previous studies have reported associations between frailty and stroke risk,^[22,23]^ and our results extend this literature in 2 important ways: by incorporating dynamic frailty transitions and by quantifying cumulative frailty exposure. We found that even when individuals experienced some degree of improvement – such as transitions from pre-frail to robust or from frail to pre-frail/robust – their stroke risk remained higher than that of those who were stably robust. At the same time, across both the overall population and frailty level-stratified analyses, worsening frailty was uniformly associated with higher risk, whereas improvement consistently reduced risk. These observations highlight frailty as a dynamic and accumulative exposure rather than a fixed health attribute. By constructing a cumulative FI and examining it both continuously and categorically, we demonstrated that each 1 − SD increase in cumulative FI conferred a 55% increase in stroke risk, and individuals in the highest quartile had a 229% higher risk than those in the lowest quartile. From a pathophysiological perspective, cumulative frailty burden reflects persistent multisystem physiological decline – a key mechanism linking frailty to cerebrovascular events. Dysregulation of the hypothalamic–pituitary–adrenal axis leads to abnormal cortisol rhythms, impaired protein synthesis, and enhanced catabolism^[24]^; declines in the growth hormone–insulin-like growth factor-1 axis further exacerbate sarcopenia and metabolic dysfunction^[25]^; chronic systemic inflammation promotes endothelial dysfunction, atherosclerosis, and neuroinflammation^[26]^; mitochondrial dysfunction limits adenosine triphosphate production and compromises antioxidant capacity and muscle regeneration^[27]^; while insulin resistance^[28]^ and dyslipidemia^[29]^ create a pro-atherogenic metabolic environment. The accumulation and interaction of these multisystem deficits drive the progression from frailty to overt cerebrovascular pathology.

Importantly, participants who transitioned from pre-frailty to robustness had a lower risk of incident stroke than those who remained pre-frail, and their risk was not statistically different from that of participants who remained robust. This pattern is consistent with the concept that pre-frailty may represent a potentially reversible stage, in which preserved physiological reserve and compensatory capacity may mitigate vascular vulnerability. Potential mechanisms may include more favorable inflammatory and oxidative stress profiles, including preserved antioxidant capacity, which could help maintain endothelial and cerebrovascular homeostasis.^[30]^ These findings support pre-frailty as a clinically relevant window for early intervention, including optimization of cardiovascular risk factors, improvement in dietary quality, and regular physical activity.^[31,32]^ Nevertheless, this observation should be interpreted cautiously. Apparent recovery from pre-frailty to robustness may not necessarily indicate complete physiological restoration, but may partly reflect measurement variability in FI components, regression to the mean, or short-term fluctuations in self-reported health and functional status. Residual confounding and the limited statistical power inherent to analyses based on multiple frailty-transition categories, particularly for detecting modest changes in stroke risk, may also have contributed to the lack of statistical significance. Therefore, the absence of a statistically significant excess stroke risk in the pre-frail to robust group should not be interpreted as definitive evidence that recovery to robustness fully normalizes stroke risk. Further studies with repeated frailty assessments across multiple waves, objective functional measures, and biomarker-based phenotyping are needed to distinguish sustained physiological recovery from transient or measurement-related changes.

We also observed that deterioration from a robust state conferred a greater increase in stroke risk than deterioration from a pre-frail state. This may be partly attributable to the presence of individuals with more severe transitions (e.g., robust directly to frail), but a more fundamental explanation lies in the physiological stability of a robust state. Robust individuals maintain highly efficient homeostatic regulation, and abrupt disruption – such as sudden deterioration to frailty – may trigger cascading physiological dysregulation, including loss of vascular elasticity, heightened coagulability, and amplified inflammatory activation,^[33]^ thereby sharply increasing stroke susceptibility. In contrast, individuals already in a pre-frail state have partial homeostatic impairment; further decline represents a more gradual accumulation of pathology rather than an acute destabilization, resulting in a more modest risk increment. These findings reinforce the importance of early detection and early intervention.

Subgroup analyses showed broadly consistent associations across strata, with 1 exception: among participants who were robust at baseline, the excess stroke risk associated with transition to pre-frail/frail status was attenuated in those with heart disease. This finding should not be interpreted as suggesting a protective effect of cardiac disease. Rather, individuals with established heart disease may be more likely to receive regular medical follow-up and active cardiovascular risk-factor management, including blood pressure control, lipid-lowering therapy, antiplatelet treatment, and anticoagulation when clinically indicated, which may partially mitigate the additional cerebrovascular risk associated with frailty deterioration.^[34–38]^ In addition, patients with heart disease may already have a higher baseline vascular risk, making the relative excess risk attributable to frailty progression appear smaller. Heart disease, particularly atrial fibrillation, may also modulate the frailty–stroke relationship through cardioembolic risk, inflammation-related prothrombotic pathways, multimorbidity, impaired mobility, and reduced physiological reserve. Prior studies in elderly populations have further shown that atrial fibrillation and other cardiac variables are associated with long-term and in-hospital mortality, supporting the clinical relevance of incorporating cardiac comorbidity into frailty-based risk stratification.^[39–41]^ These mechanisms suggest that frailty-related stroke risk may be partly shaped by the underlying cardiac risk profile. Incorporating cardiac risk-related variables into the cumulative FI, or integrating them alongside the FI, may further improve its predictive validity and clinical utility in older patients. In particular, cardiac-specific characteristics such as atrial fibrillation burden, rhythm status, anticoagulation quality, treatment adherence, and longitudinal risk-factor control may capture important dimensions of cardioembolic and vascular vulnerability that are not fully reflected by conventional frailty measures. Future studies with detailed cardiac phenotyping are therefore warranted to determine whether cardiac-specific variables can enhance frailty-based risk stratification and support more individualized stroke prevention in older adults.

Exploratory age-stratified analyses showed broadly consistent directions of association between frailty transitions and stroke risk across age groups. However, among participants aged ≥75 years, most risk estimates were attenuated and did not reach statistical significance, with wider CIs. This pattern is likely explained, at least in part, by the smaller number of participants and stroke events in this subgroup. Because the frailty-transition framework further classifies participants into multiple discrete categories, the number of participants and events within each category is further reduced, thereby limiting statistical power. Similarly, although the cumulative FI showed generally consistent predictive performance across age groups, categorization into quartiles also led to attenuated and mostly nonsignificant risk estimates, accompanied by wider CIs. These findings highlight the need for validation in larger cohorts enriched for older and very old adults to obtain more stable estimates of frailty-related stroke risk. Beyond issues of statistical power, our findings also suggest potential limitations of applying uniform FI cutoffs derived from the overall population to older adults. The same absolute FI value may have different clinical implications across age groups.^[42]^ For instance, an FI of 0.3 in a younger individual may indicate an unusually high accumulation of health deficits relative to age peers, whereas the same FI value in an older individual may more closely reflect the deficit burden commonly observed at advanced ages. Thus, although uniform FI cutoffs facilitate comparability across age groups, they may not fully capture the heterogeneity and risk gradient of frailty within older populations. Future studies with sufficient representation of older and very old adults should compare uniform FI cutoffs with age-specific or age-referenced thresholds for stroke risk prediction, thereby identifying frailty assessment strategies better suited to aging populations. This need for age-tailored risk stratification is supported by evidence from elderly nonelective surgical cohorts, in which frailty-derived scoring models improved prediction of perioperative myocardial injury, suggesting that frailty-based approaches may enhance risk assessment when conventional models perform suboptimally in older adults.^[43]^

### 4.1. Clinical implications

By integrating whole-population models with frailty-level-stratified analyses, this study establishes clear risk gradients for different frailty-transition patterns and strengthens the role of frailty assessment in primary stroke prevention. Furthermore, the robust linear association between cumulative frailty burden and incident stroke, supported by multiple sensitivity analyses, highlights cumulative FI as a powerful and clinically actionable risk indicator. These results suggest that frailty screening should be incorporated into routine health assessments, even among individuals who appear robust, to identify high-risk trajectories at an early stage. Incorporating longitudinal frailty monitoring into public health systems and leveraging electronic health records for dynamic risk stratification may enable earlier, more personalized preventive strategies. Beyond stroke prediction, the FI may also be relevant to mortality risk standardization by reflecting baseline physiological reserve and multisystem deficit burden beyond age and comorbidity alone. However, this possibility could not be directly evaluated in the present study because only 5 deaths occurred during follow-up, providing insufficient event numbers for reliable mortality modeling or risk standardization analyses. Validation in cohorts with adequate mortality events is therefore warranted.

### 4.2. Limitations

This study has several limitations. First, stroke events were based on self-reported physician diagnoses, and stroke subtypes, including ischemic and hemorrhagic stroke, could not be distinguished. Future studies using medical records, neuroimaging, or registry-based validation are needed to assess subtype-specific outcomes. Second, residual confounding cannot be excluded despite adjustment for multiple covariates and sensitivity analyses, particularly from unmeasured factors such as medication use, vascular risk-factor control, diet, and healthcare access. Third, reverse causality remains possible, as some FI components may reflect early manifestations of subclinical cerebrovascular disease, although analyses excluding prevalent and early incident stroke showed consistent results. Fourth, cumulative frailty burden was assessed using only 2 baseline waves, which may not fully capture long-term frailty trajectories. Fifth, the relatively young mean age of the analytic sample may limit the generalizability of our findings to the oldest-old population, particularly individuals aged ≥80 years. Although exploratory age-stratified analyses were conducted (<75 vs ≥75 years), the limited sample size and consequent reduction in statistical power resulted in nonsignificant risk estimates in most frailty transition groups; therefore, these findings should be interpreted with caution. Sixth, death before stroke may represent a competing event; however, only 5 deaths occurred, precluding reliable competing-risk modeling. Future studies with more mortality events should apply competing-risk or multistate models. Finally, although CHARLS is nationally representative of middle-aged and older Chinese adults, external validation in independent and more diverse cohorts is warranted.

## 5. Conclusion

Dynamic changes in frailty status and cumulative frailty burden are strongly associated with the risk of stroke. Incorporating frailty monitoring – including both transition patterns and cumulative FI – into routine clinical assessments may facilitate earlier identification of high-risk individuals and enable more timely and targeted preventive interventions.

## Acknowledgments

We extend our deepest gratitude to the study participants and the members of the CHARLS cohort.

## Author contributions

**Conceptualization:** Hui Li, Chuanyong Cui, Puchen Hong.

**Data curation:** Hui Li.

**Formal analysis:** Hui Li, Chuanyong Cui.

**Funding acquisition:** Bi Tang.

**Investigation:** Chuanyong Cui, Puchen Hong.

**Methodology:** Hui Li, Chuanyong Cui.

**Project administration:** Bi Tang.

**Resources:** Bi Tang.

**Software:** Chuanyong Cui, Bi Tang.

**Supervision:** Bi Tang.

**Validation:** Bi Tang.

**Writing – original draft:** Hui Li, Chuanyong Cui.

**Writing – review & editing:** Hui Li, Bi Tang.




































