# Supplementary figures and images for: Dynamic frailty changes, cumulative frailty index, and the risk of stroke: Evidence from the China health and retirement longitudinal study
Source: Medicine (Baltimore). 2026 Jul 10;105(28):e49726. doi: 10.1097/MD.0000000000049726 (PMC13363272; doi:10.1097/MD.0000000000049726)

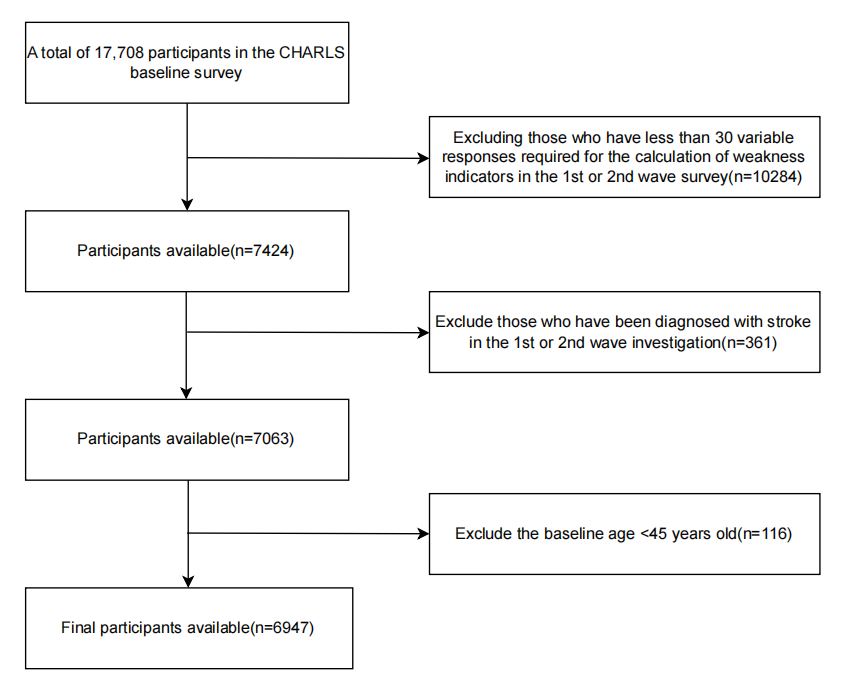

Supplement: Supplementary file 1 [file medi-105-e49726-s001.tif]

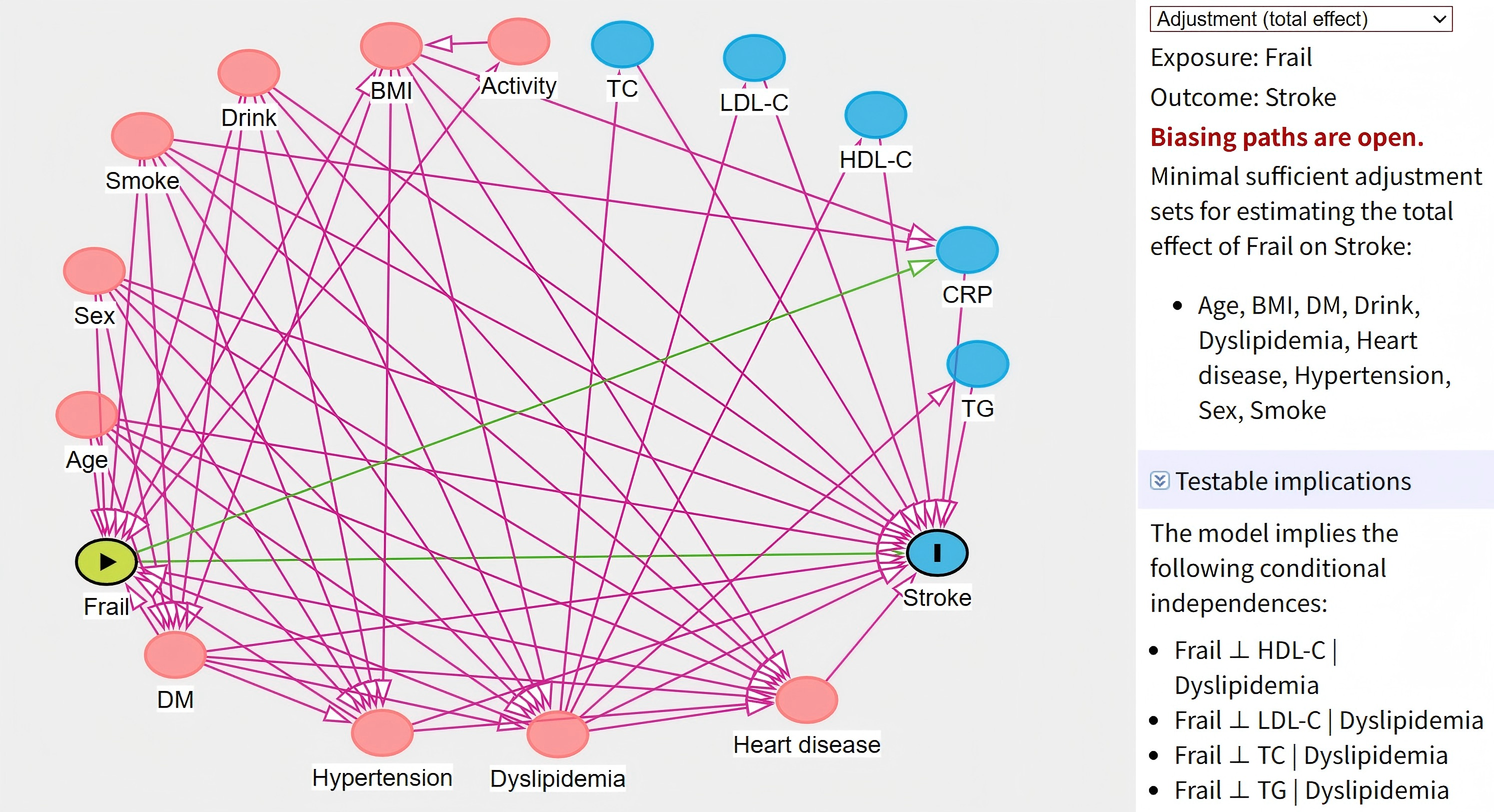

Supplement: Supplementary file 2 [file medi-105-e49726-s002.tif]

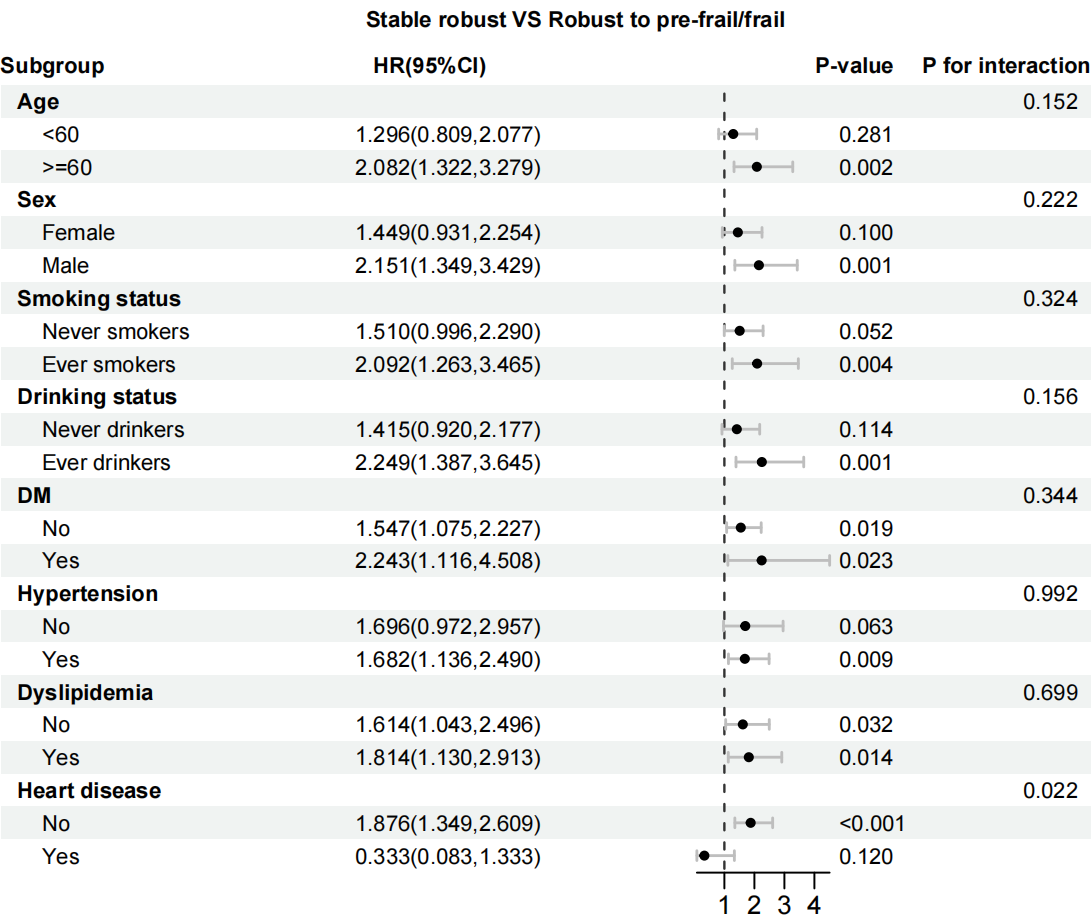

Supplement: Supplementary file 3 [file medi-105-e49726-s003.tif]

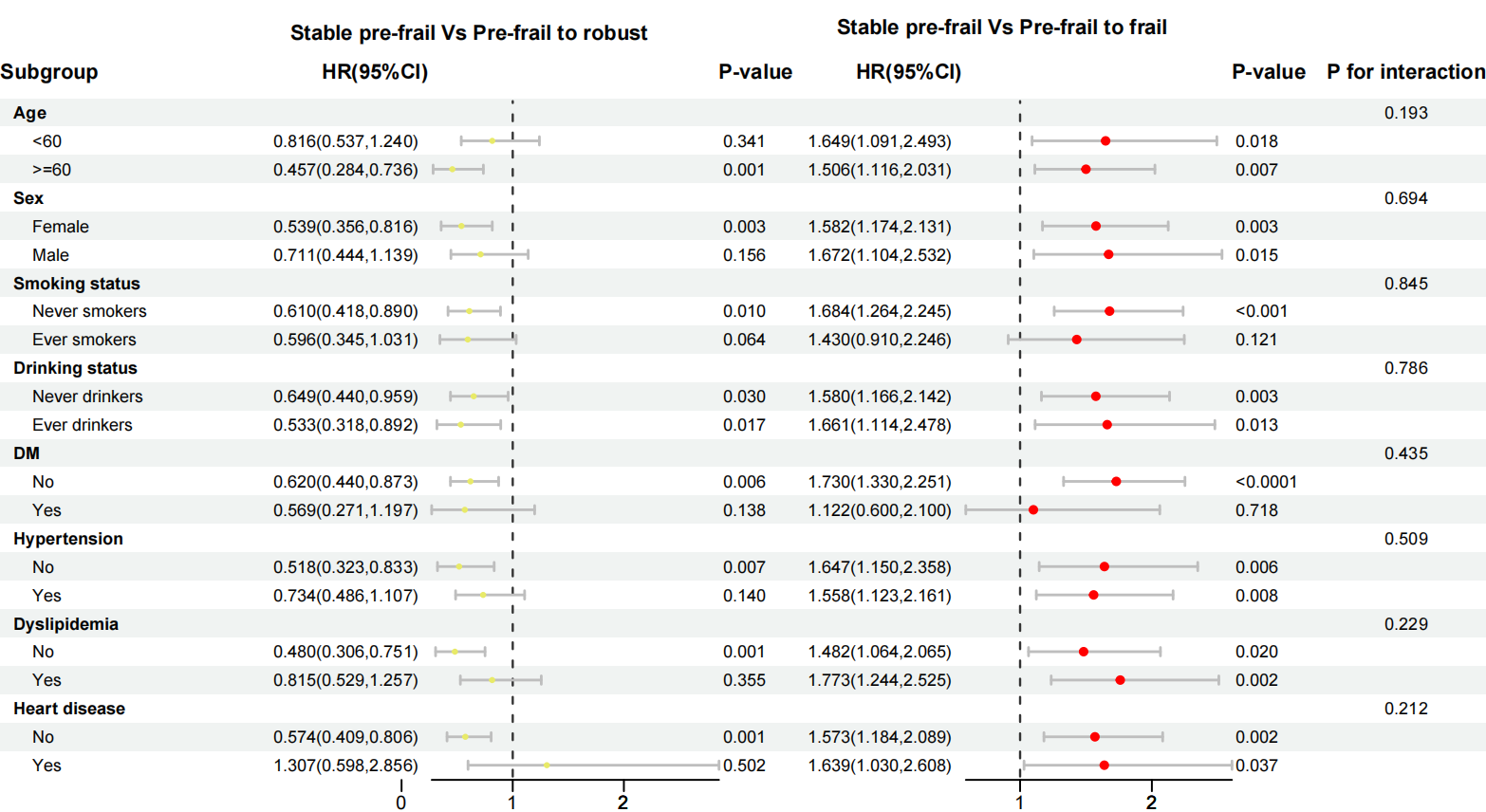

Supplement: Supplementary file 4 [file medi-105-e49726-s004.tif]

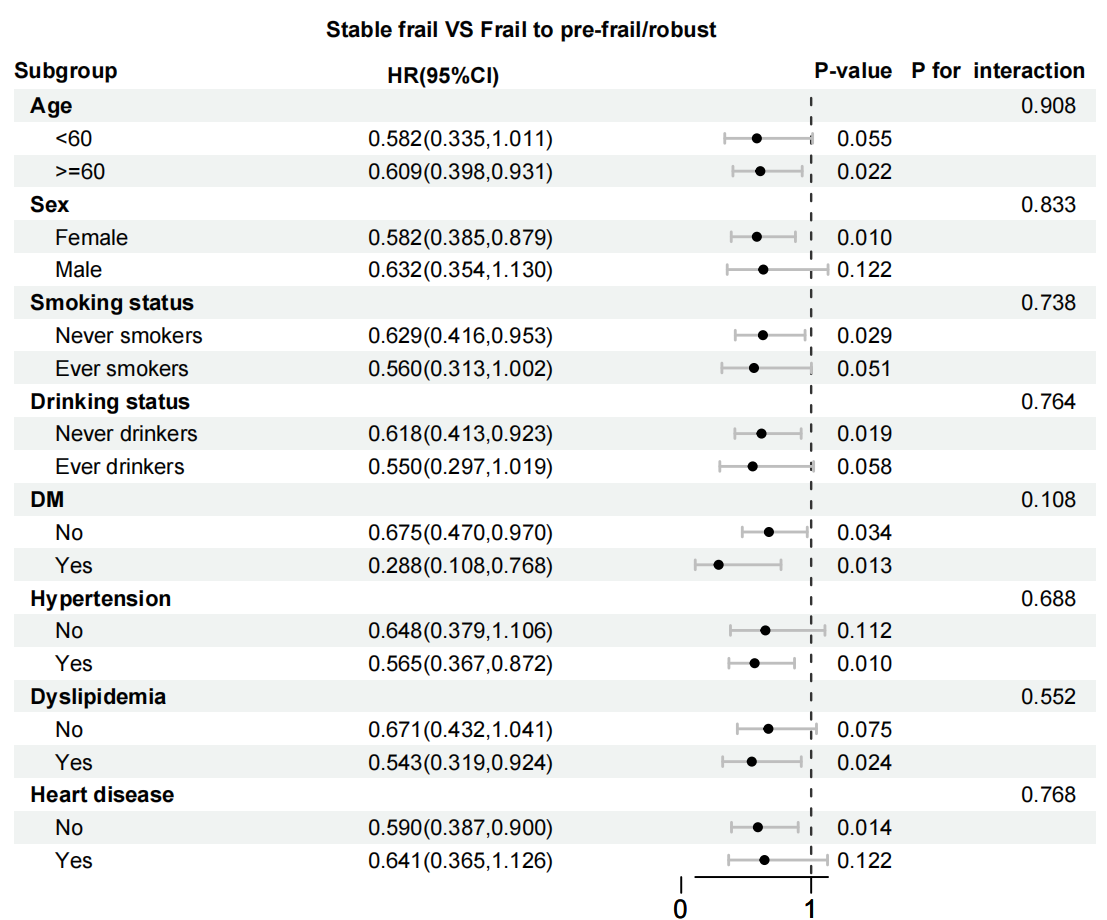

Supplement: Supplementary file 5 [file medi-105-e49726-s005.tif]
