# Supplementary material for: Dynamic frailty changes, cumulative frailty index, and the risk of stroke: Evidence from the China health and retirement longitudinal study
Source: Medicine (Baltimore). 2026 Jul 10;105(28):e49726. doi: 10.1097/MD.0000000000049726 (PMC13363272; doi:10.1097/MD.0000000000049726)
Supplement: Supplementary file 6 [file medi-105-e49726-s006.docx]

**Table S1.** List of health deficits items included in the frailty index.

| **Type of deficit** | **Item** | **Variables** | **Cut-off point** |
| --- | --- | --- | --- |
| Activities of daily living | 1 | Bathing | No=0; a little =0.33; need help = 0.67; Yes=1 |
|  | 2 | Dressing | No=0; a little =0.33; need help = 0.67; Yes=1 |
|  | 3 | Use of toilet | No=0; a little =0.33; need help = 0.67; Yes=1 |
|  | 4 | Transferring | No=0; a little =0.33; need help = 0.67; Yes=1 |
|  | 5 | Continence | No=0; a little =0.33; need help = 0.67; Yes=1 |
|  | 6 | Eating | No=0; a little =0.33; need help = 0.67; Yes=1 |
| Instrumental  activities of daily living | 7 | Cooking | No=0; a little =0.33; need help = 0.67; Yes=1 |
|  | 8 | Shopping | No=0; a little =0.33; need help = 0.67; Yes=1 |
|  | 9 | Doing household | No=0; a little =0.33; need help = 0.67; Yes=1 |
|  | 10 | Taking medicine | No=0; a little =0.33; need help = 0.67; Yes=1 |
|  | 11 | Managing money | No=0; a little =0.33; need help = 0.67; Yes=1 |
| Physical functional limitations | 12 | Lift a weight of 5 kg | No=0; a little =0.33; need help = 0.67; Yes=1 |
|  | 13 | Walking 1 km | No=0; a little =0.33; need help = 0.67; Yes=1 |
|  | 14 | Walking 100m | No=0; a little =0.33; need help = 0.67; Yes=1 |
|  | 15 | Stooping, kneeling, or crouching | No=0; a little =0.33; need help = 0.67; Yes=1 |
|  | 16 | Able to stand up from sitting | No=0; a little =0.33; need help = 0.67; Yes=1 |
|  | 17 | Able to pick up a coin from a table | No=0; a little =0.33; need help = 0.67; Yes=1 |
|  | 18 | Running or jogging about 1 km | No=0; a little =0.33; need help = 0.67; Yes=1 |
|  | 19 | Reaching or extend arms | No=0; a little =0.33; need help = 0.67; Yes=1 |
|  | 20 | Climbing several flights of stairs without resting | No=0; a little =0.33; need help = 0.67; Yes=1 |
| Chronic disease | 21 | Chronic lung diseases (Chronic bronchitis, emphysema) | Yes=1; no=0 |
|  | 22 | Asthma | Yes=1; no=0 |

|  | 23 | stroke | Yes=1; no=0 |
| --- | --- | --- | --- |
|  | 24 | CVD | Yes=1; no=0 |
|  | 25 | Gastric or duodenal ulcer | Yes=1; no=1 |
|  | 26 | Kidney disease | Yes=1; no=1 |
|  | 27 | Liver disease | Yes=1; no=1 |
|  | 28 | Memory related disease (Dementia, brain atrophy, and  Parkinson's disease) | Yes=1; no=0 |
|  | 29 | Emotional, nervous, or psychiatric problems | Yes=1; no=0 |
| Mental health | 30 | Feel depressed | Always=1; often=0.67; sometimes=0.33; seldom or never=0 |
|  | 31 | Feel fearful | Always=1; often=0.67; sometimes=0.33; seldom or never=0 |
|  | 32 | Feel happy | Always=0; often=0.33; sometimes=0.67; seldom or never=1 |
|  | 33 | Feel everything was an effort | Always=1; often=0.67; sometimes=0.33; seldom or never=0 |
|  | 34 | Feel could not get "going" | Always=1; often=0.67; sometimes=0.33; seldom or never=0 |
| Subjective  functioning | 35 | Self-rated health | Very good=0; good=0.25; average=0.5; bad=0.75; very bad=1 |
