# Supplementary material for: Dynamic frailty changes, cumulative frailty index, and the risk of stroke: Evidence from the China health and retirement longitudinal study
Source: Medicine (Baltimore). 2026 Jul 10;105(28):e49726. doi: 10.1097/MD.0000000000049726 (PMC13363272; doi:10.1097/MD.0000000000049726)
Supplement: Supplementary file 7 [file medi-105-e49726-s007.docx]

| **Table S2. Associations of the Frail State Transition Pattern with Stroke, evaluated using the Cox Proportional Hazards Model in the whole cohort.** | | | | | | |
| --- | --- | --- | --- | --- | --- | --- |
|  | **Crude model** | | **Model 1** | | **Model 2** | |
| **Exposure** | **HR (95% CI)** | ***P*-value** | **HR (95% CI)** | ***P*-value** | **HR (95% CI)** | ***P*-value** |
|  |  |  |  |  |  |  |
| Stable robust | Ref. |  | Ref. |  | Ref. |  |
| Pre-frail to robust | 1.31(0.91,1.90) | 0.15 | 1.38(0.95,2.00) | 0.09 | 1.41(0.97,2.04) | 0.07 |
| Robust to pre-frail/frail | 1.72(1.25,2.37) | <0.001 | 1.76(1.28,2.43) | <0.001 | 1.73(1.26,2.39) | <0.001 |
| Stable pre-frail | 2.16(1.66,2.83) | <0.001 | 2.25(1.72,2.95) | <0.001 | 2.19(1.67,2.87) | <0.001 |
| Frail to pre-frail/robust | 2.78(1.94,3.98) | <0.001 | 2.77(1.93,3.99) | <0.001 | 2.71(1.87,3.91) | <0.001 |
| Pre-frail to frail | 3.40(2.48,4.67) | <0.001 | 3.52(2.55,4.84) | <0.001 | 3.46(2.51,4.78) | <0.001 |
| Stable frail | 4.77(3.51,6.48) | <0.001 | 4.60(3.36,6.29) | <0.001 | 4.38(3.17,6.06) | <0.001 |
| P for trend |  | <0.001 |  | <0.001 |  | <0.001 |
| Crudel model: No covariates were adjusted | |  |  |  |  |  |
| model 1: Age, sex, BMI, smoking status, drinking status, marital status, education, CRP, HDL-C, HbA1c, mean sbp, mean dbp, activity | | | | | |  |
| model 2: Age, sex, BMI, smoking status, drinking status, marital status, education, CRP, HDL-C, HbA1c, mean sbp, mean dbp, activity, DM, hypertension, dyslipidemia, heart disease | | | | | | |
| CRP:C-reactive protein;HbA1c:Hemoglobin A1c | |  |  |  |  |  |
