# Supplementary material for: Dynamic frailty changes, cumulative frailty index, and the risk of stroke: Evidence from the China health and retirement longitudinal study
Source: Medicine (Baltimore). 2026 Jul 10;105(28):e49726. doi: 10.1097/MD.0000000000049726 (PMC13363272; doi:10.1097/MD.0000000000049726)
Supplement: Supplementary file 9 [file medi-105-e49726-s009.docx]

| **Table S4. Associations of the Cumulative FI with Stroke, evaluated using the Cox Proportional Hazards Model in the whole cohort.** | | | | | | |
| --- | --- | --- | --- | --- | --- | --- |
|  | **Crude model** | | **Model 1** | | **Model 2** | |
| **Exposure** | **HR (95% CI)** | **P-value** | **HR (95% CI)** | **P-value** | **HR (95% CI)** | **P-value** |
|  |  |  |  |  |  |  |
| **Per 1‑SD increase** | 1.57(1.48,1.67) | <0.001 | 1.55(1.45,1.65) | <0.001 | 1.54(1.44,1.64) | <0.001 |
| **Quatipartiple group** |  |  |  |  |  |  |
| *Q1* | Ref. |  | Ref. |  | Ref. |  |
| *Q2* | 1.37(1.06,1.77) | 0.02 | 1.42(1.10,1.84) | 0.01 | 1.39(1.07,1.81) | 0.01 |
| *Q3* | 1.96(1.54,2.49) | <0.001 | 2.03(1.59,2.59) | <0.001 | 1.99(1.55,2.54) | <0.001 |
| *Q4* | 3.43(2.73,4.31) | <0.001 | 3.40(2.69,4.29) | <0.001 | 3.28(2.58,4.17) | <0.001 |
| P for trend |  | <0.001 |  | <0.001 |  | <0.001 |
| P for trend(Median value) |  | <0.001 |  | <0.001 |  | <0.001 |
| Crudel model: No covariates were adjusted | |  |  |  |  |  |
| model 1: Age, sex, BMI, smoking status, drinking status, marital status, education, CRP, HDL-C, HbA1c, mean sbp, mean dbp, activity | | | | | |  |
| model 2: Age, sex, BMI, smoking status, drinking status, marital status, education, CRP, HDL-C, HbA1c, mean sbp, mean dbp, activity, DM, hypertension, dyslipidemia, heart disease | | | | | | |
| CRP:C-reactive protein;HbA1c:Hemoglobin A1c | |  |  |  |  |  |
