# Supplementary material for: Dynamic frailty changes, cumulative frailty index, and the risk of stroke: Evidence from the China health and retirement longitudinal study
Source: Medicine (Baltimore). 2026 Jul 10;105(28):e49726. doi: 10.1097/MD.0000000000049726 (PMC13363272; doi:10.1097/MD.0000000000049726)
Supplement: Supplementary file 10 [file medi-105-e49726-s010.docx]

| **Table S5. Associations of the Frail State Transition Pattern with Stroke, evaluated using the Cox Proportional Hazards Model in the whole cohort.** | | | | | | |
| --- | --- | --- | --- | --- | --- | --- |
|  | **Crude model** | | **Model 1** | | **Model 2** | |
| **Exposure** | **HR (95% CI)** | ***P*-value** | **HR (95% CI)** | ***P*-value** | **HR (95% CI)** | ***P*-value** |
|  |  |  |  |  |  |  |
| Stable robust | Ref. |  | Ref. |  | Ref. |  |
| Pre-frail to robust | 1.36(0.92,2.01) | 0.13 | 1.35(0.91,2.00) | 0.13 | 1.42(0.96,2.11) | 0.08 |
| Robust to pre-frail/frail | 1.85(1.32,2.60) | <0.001 | 1.87(1.33,2.63) | <0.001 | 1.85(1.32,2.60) | <0.001 |
| Stable pre-frail | 2.23(1.68,2.97) | <0.001 | 2.24(1.69,2.99) | <0.001 | 2.22(1.66,2.96) | <0.001 |
| Frail to pre-frail/robust | 2.45(1.64,3.66) | <0.001 | 2.45(1.63,3.67) | <0.001 | 2.38(1.58,3.58) | <0.001 |
| Pre-frail to frail | 3.28(2.32,4.62) | <0.001 | 3.32(2.35,4.70) | <0.001 | 3.29(2.32,4.67) | <0.001 |
| Stable frail | 4.52(3.24,6.33) | <0.001 | 4.43(3.15,6.23) | <0.001 | 4.25(2.99,6.04) | <0.001 |
| P for trend |  | <0.001 |  | <0.001 |  | <0.001 |
| Crudel model: No covariates were adjusted | |  |  |  |  |  |
| Model 1: Age, sex, smoking status, drinking status, BMI | | |  |  |  |  |
| Model 2: Age, sex, smoking status, drinking status, BMI, DM, hypertension, dyslipidemia, heart disease | | | | |  |  |
