# Supplementary material for: Dynamic frailty changes, cumulative frailty index, and the risk of stroke: Evidence from the China health and retirement longitudinal study
Source: Medicine (Baltimore). 2026 Jul 10;105(28):e49726. doi: 10.1097/MD.0000000000049726 (PMC13363272; doi:10.1097/MD.0000000000049726)
Supplement: Supplementary file 11 [file medi-105-e49726-s011.docx]

| **Table S6. Associations of the Frail State Transition Pattern with Stroke, evaluated using the Cox Proportional Hazards Model in the group of people.** | | | | | | |
| --- | --- | --- | --- | --- | --- | --- |
|  | **Crude model** | | **Model 1** | | **Model 2** | |
| **Exposure** | **HR (95% CI)** | ***P*-value** | **HR (95% CI)** | ***P*-value** | **HR (95% CI)** | ***P*-value** |
|  |  |  |  |  |  |  |
| **The first group** |  |  |  |  |  |  |
| *Stable robust* | Ref. |  | Ref. |  | Ref. |  |
| *Robust to pre-frail/frail* | 1.85(1.32,2.59) | <0.001 | 1.87(1.33,2.62) | <0.001 | 1.97(1.40,2.78) | <0.001 |
| **The second group** |  |  |  |  |  |  |
| *Stable pre-frail* | Ref. |  | Ref. |  | Ref. |  |
| *Pre-frail to robust* | 0.61(0.44,0.84) | 0.003 | 0.60(0.44,0.84) | 0.002 | 0.65(0.47,0.90) | 0.01 |
| *Pre-frail to frail* | 1.47(1.13,1.92) | 0.004 | 1.47(1.12,1.91) | 0.005 | 1.47(1.13,1.92) | 0.005 |
| **The third group** |  |  |  |  |  |  |
| *Stable frail* | Ref. |  | Ref. |  | Ref. |  |
| *Frail to pre-frail/robust* | 0.54(0.37,0.79) | 0.002 | 0.55(0.37,0.80) | 0.002 | 0.56(0.38,0.82) | 0.003 |
| Crudel model: No covariates were adjusted | |  |  |  |  |  |
| Model 1: Age, sex, smoking status, drinking status, BMI | |  |  |  |  |  |
| Model 2: Age, sex, smoking status, drinking status, BMI,DM, hypertension, dyslipidemia, heart disease | | | |  |  |  |
