# Supplementary material for: Dynamic frailty changes, cumulative frailty index, and the risk of stroke: Evidence from the China health and retirement longitudinal study
Source: Medicine (Baltimore). 2026 Jul 10;105(28):e49726. doi: 10.1097/MD.0000000000049726 (PMC13363272; doi:10.1097/MD.0000000000049726)
Supplement: Supplementary file 13 [file medi-105-e49726-s013.docx]

| **Table S8. Associations of the Frail State Transition Pattern with Stroke, evaluated using the Cox Proportional Hazards Model in the whole cohort.** | | | | | | |
| --- | --- | --- | --- | --- | --- | --- |
|  | **Crude model** | | **Model 1** | | **Model 2** | |
| **Exposure** | **HR (95% CI)** | ***P*-value** | **HR (95% CI)** | ***P*-value** | **HR (95% CI)** | ***P*-value** |
|  |  |  |  |  |  |  |
| Stable robust | Ref. |  | Ref. |  | Ref. |  |
| Pre-frail to robust | 1.31(0.91,1.90) | 0.15 | 1.43(0.99,2.08) | 0.06 | 1.45(1.00,2.10) | 0.05 |
| Robust to pre-frail/frail | 1.72(1.25,2.37) | <0.001 | 1.82(1.32,2.51) | <0.001 | 1.78(1.29,2.46) | <0.001 |
| Stable pre-frail | 2.17(1.66,2.83) | <0.001 | 2.30(1.76,3.01) | <0.001 | 2.24(1.71,2.94) | <0.001 |
| Frail to pre-frail/robust | 2.78(1.94,3.98) | <0.001 | 2.80(1.95,4.03) | <0.001 | 2.75(1.90,3.97) | <0.001 |
| Pre-frail to frail | 3.40(2.48,4.67) | <0.001 | 3.60(2.61,4.97) | <0.001 | 3.54(2.56,4.89) | <0.001 |
| Stable frail | 4.77(3.51,6.48) | <0.001 | 4.70(3.43,6.44) | <0.001 | 4.50(3.25,6.22) | <0.001 |
| P for trend |  | <0.001 |  | <0.001 |  | <0.001 |
| Crudel model: No covariates were adjusted | |  |  |  |  |  |
| model 1: Age, sex, smoking status, drinking status, BMI | |  |  |  |  |  |
| model 2: Age, sex, smoking status, drinking status, BMI, DM, hypertension, dyslipidemia, heart disease | | | |  |  |  |
