# Supplementary material for: Dynamic frailty changes, cumulative frailty index, and the risk of stroke: Evidence from the China health and retirement longitudinal study
Source: Medicine (Baltimore). 2026 Jul 10;105(28):e49726. doi: 10.1097/MD.0000000000049726 (PMC13363272; doi:10.1097/MD.0000000000049726)
Supplement: Supplementary file 15 [file medi-105-e49726-s015.docx]

| **Table S10. Associations of the Cumulative FI with Stroke, evaluated using the Cox Proportional Hazards Model in the whole cohort.** | | | | | | |
| --- | --- | --- | --- | --- | --- | --- |
|  | **Crude model** | | **Model 1** | | **Model 2** | |
| **Exposure** | **HR (95% CI)** | **P-value** | **HR (95% CI)** | **P-value** | **HR (95% CI)** | **P-value** |
|  |  |  |  |  |  |  |
| **Per 1‑SD increase** | 1.54(1.44,1.64) | <0.001 | 1.57(1.47,1.67) | <0.001 | 1.55(1.45,1.65) | <0.001 |
| **Quatipartiple group** |  |  |  |  |  |  |
| *Q1* | Ref. |  | Ref. |  | Ref. |  |
| *Q2* | 1.39(1.07,1.80) | 0.01 | 1.40(1.09,1.81) | 0.01 | 1.39(1.07,1.80) | 0.01 |
| *Q3* | 1.98(1.56,2.52) | <0.001 | 2.00(1.56,2.56) | <0.001 | 1.99(1.56,2.54) | <0.001 |
| *Q4* | 3.46(2.75,4.34) | <0.001 | 3.47(2.75,4.34) | <0.001 | 3.33(2.63,4.23) | <0.001 |
| P for trend |  | <0.001 |  | <0.001 |  | <0.001 |
| P for trend(Median value) |  | <0.001 |  | <0.001 |  | <0.001 |
| Crudel model: No covariates were adjusted |  |  |  |  |  |  |
| Model 1: Age, sex, smoking status, drinking status, BMI | |  |  |  |  |  |
| Model 2: Age, sex, smoking status, drinking status, BMI, DM, hypertension, dyslipidemia, heart disease | | | | | | |
