# Supplementary material for: Dynamic frailty changes, cumulative frailty index, and the risk of stroke: Evidence from the China health and retirement longitudinal study
Source: Medicine (Baltimore). 2026 Jul 10;105(28):e49726. doi: 10.1097/MD.0000000000049726 (PMC13363272; doi:10.1097/MD.0000000000049726)
Supplement: Supplementary file 16 [file medi-105-e49726-s016.docx]

| **Table S11. Associations of Frail State Transition Pattern with Stroke by Age <75 and >=75 Years, evaluated using the Cox Proportional Hazards Model.** | | | | | | | | | | |
| --- | --- | --- | --- | --- | --- | --- | --- | --- | --- | --- |
| **Age group / Exposure** | | **Descriptive statistics** | | | **Crude model** | | **Model 1** | | **Model 2** | |
| **Age group** | **Exposure** | **N** | **Events** | **Proportion (%)** | **HR (95% CI)** | **P-value** | **HR (95% CI)** | **P-value** | **HR (95% CI)** | **P-value** |
| **Age <75 years** | Stable robust | 1197 | 63 | 5.3 | Ref. |  | Ref. |  | Ref. |  |
|  | Pre-frail to robust | 664 | 46 | 6.9 | 1.38 (0.94, 2.02) | 0.096 | 1.37 (0.94, 2.01) | 0.102 | 1.44 (0.99, 2.11) | 0.059 |
|  | Robust to pre-frail/frail | 881 | 79 | 9 | 1.79 (1.29, 2.49) | <0.001 | 1.80 (1.29, 2.51) | <0.001 | 1.79 (1.28, 2.49) | <0.001 |
|  | Stable pre-frail | 2198 | 246 | 11.2 | 2.24 (1.70, 2.95) | <0.001 | 2.21 (1.67, 2.91) | <0.001 | 2.20 (1.66, 2.91) | <0.001 |
|  | Frail to pre-frail/robust | 345 | 49 | 14.2 | 3.00 (2.06, 4.36) | <0.001 | 2.94 (2.02, 4.28) | <0.001 | 2.83 (1.94, 4.15) | <0.001 |
|  | Pre-frail to frail | 518 | 78 | 15.1 | 3.39 (2.43, 4.73) | <0.001 | 3.33 (2.38, 4.65) | <0.001 | 3.34 (2.38, 4.68) | <0.001 |
|  | Stable frail | 438 | 90 | 20.5 | 5.17 (3.74, 7.13) | <0.001 | 4.97 (3.59, 6.89) | <0.001 | 4.79 (3.42, 6.71) | <0.001 |
| **Age >=75 years** | Stable robust | 88 | 5 | 5.7 | Ref. |  | Ref. |  | Ref. |  |
|  | Pre-frail to robust | 55 | 2 | 3.6 | 0.57 (0.11, 2.95) | 0.504 | 0.56 (0.11, 2.90) | 0.492 | 0.67 (0.13, 3.49) | 0.635 |
|  | Robust to pre-frail/frail | 91 | 5 | 5.5 | 0.97 (0.28, 3.34) | 0.956 | 0.98 (0.28, 3.42) | 0.973 | 0.96 (0.27, 3.38) | 0.949 |
|  | Stable pre-frail | 195 | 16 | 8.2 | 1.33 (0.49, 3.64) | 0.575 | 1.35 (0.49, 3.72) | 0.566 | 1.31 (0.47, 3.68) | 0.61 |
|  | Frail to pre-frail/robust | 66 | 4 | 6.1 | 1.19 (0.32, 4.42) | 0.797 | 1.36 (0.35, 5.18) | 0.656 | 1.44 (0.38, 5.48) | 0.596 |
|  | Pre-frail to frail | 87 | 10 | 11.5 | 2.99 (1.02, 8.76) | 0.046 | 3.28 (1.11, 9.73) | 0.032 | 3.43 (1.14, 10.32) | 0.028 |
|  | Stable frail | 124 | 12 | 9.7 | 2.37 (0.83, 6.72) | 0.106 | 2.60 (0.89, 7.57) | 0.08 | 2.78 (0.94, 8.25) | 0.065 |
| Crude model: No covariates were adjusted. | | | | | | | | | | |
| Model 1: Age, sex, smoke status, drink status, BMI. | | | | | | | | | | |
| Model 2: Age, sex, smoke status, drink status, BMI, DM, hypertension, dyslipidemia, heart disease. | | | | | | | | | | |
