# Supplementary material for: Dynamic frailty changes, cumulative frailty index, and the risk of stroke: Evidence from the China health and retirement longitudinal study
Source: Medicine (Baltimore). 2026 Jul 10;105(28):e49726. doi: 10.1097/MD.0000000000049726 (PMC13363272; doi:10.1097/MD.0000000000049726)
Supplement: Supplementary file 17 [file medi-105-e49726-s017.docx]

| **Table S12. Associations of the Frail State Transition Pattern with Stroke by Age <75 and >=75 Years in the group of people, evaluated using the Cox Proportional Hazards Model.** | | | | | | | | | | |
| --- | --- | --- | --- | --- | --- | --- | --- | --- | --- | --- |
| **Age group / Exposure** | | **Descriptive statistics** | | | **Crude model** | | **Model 1** | | **Model 2** | |
| **Age group** | **Exposure** | **N** | **Events** | **Proportion (%)** | **HR (95% CI)** | **P-value** | **HR (95% CI)** | **P-value** | **HR (95% CI)** | **P-value** |
| **Age <75 years** | **The first group** |  |  |  |  |  |  |  |  |  |
|  | *Stable robust* | 1197 | 63 | 5.3 | Ref. |  | Ref. |  | Ref. |  |
|  | *Robust to pre-frail/frail* | 881 | 79 | 9 | 1.79 (1.29, 2.49) | <0.001 | 1.83 (1.31, 2.56) | <0.001 | 1.86 (1.33, 2.61) | <0.001 |
|  | **The second group** |  |  |  |  |  |  |  |  |  |
|  | *Stable pre-frail* | 2198 | 246 | 11.2 | Ref. |  | Ref. |  | Ref. |  |
|  | *Pre-frail to robust* | 664 | 46 | 6.9 | 0.62 (0.45, 0.84) | 0.003 | 0.62 (0.46, 0.86) | 0.003 | 0.67 (0.48, 0.92) | 0.012 |
|  | *Pre-frail to frail* | 518 | 78 | 15.1 | 1.52 (1.18, 1.96) | 0.001 | 1.49 (1.16, 1.93) | 0.002 | 1.51 (1.17, 1.95) | 0.002 |
|  | **The third group** |  |  |  |  |  |  |  |  |  |
|  | *Stable frail* | 438 | 90 | 20.5 | Ref. |  | Ref. |  | Ref. |  |
|  | *Frail to pre-frail/robust* | 345 | 49 | 14.2 | 0.59 (0.42, 0.83) | 0.003 | 0.60 (0.43, 0.86) | 0.005 | 0.60 (0.42, 0.85) | 0.005 |
| **Age >=75 years** | **The first group** |  |  |  |  |  |  |  |  |  |
|  | *Stable robust* | 88 | 5 | 5.7 | Ref. |  | Ref. |  | Ref. |  |
|  | *Robust to pre-frail/frail* | 91 | 5 | 5.5 | 0.97 (0.28, 3.35) | 0.961 | 0.95 (0.26, 3.54) | 0.944 | 1.13 (0.29, 4.47) | 0.857 |
|  | **The second group** |  |  |  |  |  |  |  |  |  |
|  | *Stable pre-frail* | 195 | 16 | 8.2 | Ref. |  | Ref. |  | Ref. |  |
|  | *Pre-frail to robust* | 55 | 2 | 3.6 | 0.43 (0.10, 1.86) | 0.258 | 0.43 (0.10, 1.89) | 0.264 | 0.52 (0.11, 2.35) | 0.392 |
|  | *Pre-frail to frail* | 87 | 10 | 11.5 | 2.27 (1.03, 5.01) | 0.043 | 2.32 (1.04, 5.18) | 0.04 | 2.66 (1.16, 6.08) | 0.021 |
|  | **The third group** |  |  |  |  |  |  |  |  |  |
|  | *Stable frail* | 124 | 12 | 9.7 | Ref. |  | Ref. |  | Ref. |  |
|  | *Frail to pre-frail/robust* | 66 | 4 | 6.1 | 0.50 (0.16, 1.56) | 0.236 | 0.53 (0.15, 1.82) | 0.311 | 0.55 (0.15, 1.96) | 0.355 |
|  |  |  |  |  |  |  |  |  |  |  |
| Crude model: No covariates were adjusted. | | | | | | | | | | |
| Model 1: Age, sex, smoke status, drink status, BMI. | | | | | | | | | | |
| Model 2: Age, sex, smoke status, drink status, BMI, DM, hypertension, dyslipidemia, heart disease. | | | | | | | | | | |
