# Supplementary material for: Dynamic frailty changes, cumulative frailty index, and the risk of stroke: Evidence from the China health and retirement longitudinal study
Source: Medicine (Baltimore). 2026 Jul 10;105(28):e49726. doi: 10.1097/MD.0000000000049726 (PMC13363272; doi:10.1097/MD.0000000000049726)
Supplement: Supplementary file 18 [file medi-105-e49726-s018.docx]

| **Table S13. Associations of the Cumulative FI with Stroke by Age <75 and >=75 Years, evaluated using the Cox Proportional Hazards Model.** | | | | | | | | | | |
| --- | --- | --- | --- | --- | --- | --- | --- | --- | --- | --- |
| **Age group / Exposure** | | **Descriptive statistics** | | | **Crude model** | | **Model 1** | | **Model 2** | |
| **Age group** | **Exposure** | **N** | **Events** | **Proportion (%)** | **HR (95% CI)** | **P-value** | **HR (95% CI)** | **P-value** | **HR (95% CI)** | **P-value** |
| **Age <75 years** | Per 1-SD increase | 6241 | 651 | 10.4 | 1.59 (1.49, 1.69) | <0.001 | 1.58 (1.48, 1.68) | <0.001 | 1.56 (1.46, 1.67) | <0.001 |
|  | **Quatipartiple group** |  |  |  |  |  |  |  |  |  |
|  | *Q1* | 1613 | 96 | 6 | Ref. |  | Ref. |  | Ref. |  |
|  | *Q2* | 1610 | 127 | 7.9 | 1.38 (1.06, 1.79) | 0.018 | 1.36 (1.04, 1.77) | 0.023 | 1.36 (1.04, 1.77) | 0.025 |
|  | *Q3* | 1574 | 177 | 11.2 | 1.98 (1.54, 2.54) | <0.001 | 1.96 (1.53, 2.52) | <0.001 | 1.94 (1.51, 2.50) | <0.001 |
|  | *Q4* | 1444 | 251 | 17.4 | 3.49 (2.76, 4.41) | <0.001 | 3.38 (2.66, 4.29) | <0.001 | 3.25 (2.54, 4.16) | <0.001 |
|  | P for trend |  |  |  |  | <0.001 |  | <0.001 |  | <0.001 |
|  | P for trend (Median value) |  |  |  |  | <0.001 |  | <0.001 |  | <0.001 |
| **Age >=75 years** | Per 1-SD increase | 706 | 54 | 7.6 | 1.46 (1.18, 1.80) | <0.001 | 1.52 (1.22, 1.88) | <0.001 | 1.55 (1.25, 1.94) | <0.001 |
|  | **Quatipartiple group** |  |  |  |  |  |  |  |  |  |
|  | *Q1* | 136 | 5 | 3.7 | Ref. |  | Ref. |  | Ref. |  |
|  | *Q2* | 120 | 8 | 6.7 | 1.69 (0.55, 5.16) | 0.358 | 1.91 (0.62, 5.87) | 0.26 | 1.92 (0.62, 5.94) | 0.256 |
|  | *Q3* | 163 | 14 | 8.6 | 2.17 (0.78, 6.02) | 0.138 | 2.37 (0.84, 6.69) | 0.102 | 2.27 (0.79, 6.48) | 0.126 |
|  | *Q4* | 287 | 27 | 9.4 | 3.52 (1.35, 9.14) | 0.01 | 4.25 (1.60, 11.24) | 0.004 | 4.42 (1.66, 11.76) | 0.003 |
|  | P for trend |  |  |  |  | 0.003 |  | 0.001 |  | <0.001 |
|  | P for trend (Median value) |  |  |  |  | 0.003 |  | <0.001 |  | <0.001 |
| Crude model: No covariates were adjusted. | | | | | | | | | | |
| Model 1: Age, sex, smoke status, drink status, BMI. | | | | | | | | | | |
| Model 2: Age, sex, smoke status, drink status, BMI, DM, hypertension, dyslipidemia, heart disease. | | | | | | | | | | |
